# Clinicopathological, Radiological, and Molecular Features of Primary Lung Adenocarcinoma with Morule-Like Components

**DOI:** 10.1155/2021/9186056

**Published:** 2021-06-12

**Authors:** Li-Li Wang, Li Ding, Peng Zhao, Jing-Jing Guan, Xiao-Bin Ji, Xiao-Li Zhou, Shi-Hong Shao, Yu-Wei Zou, Wei-Wei Fu, Dong-Liang Lin

**Affiliations:** ^1^Department of Pathology, The Affiliated Hospital of Qingdao University, Qingdao, China; ^2^Medical Affairs Department, The Affiliated Hospital of Qingdao University, Qingdao, China

## Abstract

**Background:**

Morule-like component (MLC) was a rare structure in primary lung adenocarcinoma. We aimed to reveal the clinicopathological, radiological, immunohistochemical, and molecular features of lung adenocarcinoma with MLCs.

**Methods:**

Twenty lung adenocarcinomas with MLCs were collected, and computed tomographic and histological documents were reviewed. Immunohistochemistry, targeted next-generation sequencing, and Sanger sequencing for *β-catenin* gene were performed.

**Results:**

There were 9 lepidic adenocarcinomas, 8 acinar adenocarcinomas, 2 papillary adenocarcinomas, and 1 minimally invasive adenocarcinoma. Most patients (16/17) were shown a pure solid nodule, and 1 patient was shown a partly solid nodule on chest computed tomography (CT). Nine cases were accompanied with micropapillary components, and 3 were with cribriform components in which 2 suffered a worse prognosis. No significant association was found between the MCLs and the overall survival of lung adenocarcinoma (*P* = 0.109). The MLCs were often arranged in whorled or streaming patterns. The cells in MLCs showed syncytial and mild appearance. The MLCs were positive for E-cadherin, CK7, TTF-1, napsin-A, vimentin, and *β*-catenin (membrane), and negative for CK5/6, p40, p63, Synaptophysin, chromogranin A, and Cdx-2. *EGFR* mutation, *ALK-EML4* fusion, *HER*2 amplification, and *PIK3CA* mutation were detected in 16 cases, 2 cases, 1 case, and 1 case, respectively. *EGFR* mutation was more frequent in adenocarcinomas with MLCs than those without MLCs (*P* = 0.040). *β-catenin* gene mutation was not detected in any patients.

**Conclusions:**

MLC is often observed in the background of acinar, lepidic, and papillary adenocarcinomas. Lung adenocarcinomas with MLCs tend to appear as a solid mass on CT and harbor *EGFR* gene mutations. The micropapillary components and cribriform components may cause poor prognosis of lung adenocarcinomas with MLCs. Vimentin is always positive in MLCs, and it is a useful marker for the identification of MLCs.

## 1. Introduction

In the recent World Health Organization (WHO) classification of primary lung neoplasm, primary lung adenocarcinomas were subdivided into five subtypes (lepidic adenocarcinoma, papillary adenocarcinoma, micropapillary adenocarcinoma, acinar adenocarcinoma, and solid adenocarcinoma) and four uncommon variants (mucinous adenocarcinoma, colloid adenocarcinoma, fetal adenocarcinoma, and enteric adenocarcinoma) depending on their architectural and cellular features [1]. However, lung adenocarcinoma combined with morule-like components (MLCs) is not mentioned in this edition. Fornelli et al. first released a case of lung adenocarcinoma with MLCs in 2003 [2]. Until now, there have been only 35 cases reported in the English literature [2–8]. Traditional morules often demonstrated refined cell clusters composed of spindle- to oval-shaped cells, and they lack cellular atypia or mitotic figures, which have been reported in endometrial and ovarian lesions [9–11], papillary thyroid carcinoma (cribriform/morular variant) [12], and some colonic adenomas [13]. In lung tumors, the morule is one of the most important histological characteristics of pulmonary blastoma and low-grade fetal adenocarcinoma [1]. The signal pathway of Wnt/beta-catenin is usually activated in the above-mentioned morule-related tumors, in which abnormal nuclear/cytoplasmic *β*-catenin expression is often seen. Interestingly, lung adenocarcinoma with MLCs usually had no mutation in the *β-catenin* (*CTNNB1*) gene or any aberrant *β*-catenin expression [5]. Here, we report 20 cases of lung adenocarcinoma with MLCs. We attempt to reveal the clinicopathological, radiological, immunohistochemical, and molecular features of primary lung adenocarcinoma with MLCs.

## 2. Materials and Methods

### 2.1. Clinicopathological and Radiological Data

A total of 721 patients, who underwent initial surgical resection dating from 2016 to 2018, were reviewed in our study. The patients involved in our work had never received chemotherapy or radiotherapy before surgery. The median time of follow-up was 34.1 months (range from 1 to 45 months). The cases with MLCs of more than 5% were included in this study. According to this criterion, twenty patients with MLCs were selected in our work. The clinical documents and computed tomographic (CT) imaging were reviewed. This work was approved by the Ethics Committee in our Hospital on Dec. 20, 2019 (No. 25849), and all patients signed informed consent.

### 2.2. Immunohistochemistry

Immunohistochemical stainings were carried out on paraffin-embedded tissue fixed by formalin. The following antibodies were involved in our work: cytokeratin (CK) 7 (clone: OV-TL12/30), thyroid transcription factor-1 (TTF-1) (clone: SPT24), p40 (clone: BC28), CK5/6 (clone: D5/16B4), p63 (clone: 4A4), ALK (clone: D5F3), Synaptophysin (clone: SP11), chromogranin A (clone: MX018), CD56 (clone: MX039), vimentin (clone: V9), vimentin (clone: EP21), E-cadherin (clone:4A2C7), *β*-catenin (clone: 17C2), Cdx-2 (clone: EPR2764Y), napsin-A (clone: 1P64), and Ki-67 (clone: MIB1). ALK (D5F3) and vimentin were performed on an automated platform (Benchmark® XT, Ventana). The other stainings were performed on the platform of BOND-MAXTM. Immunohistochemical staining was individually evaluated by two independent pathologists. Both MLCs and the adjacent tumor area of the adenocarcinoma were assessed in the wholes slide.

### 2.3. Targeted Next-Generation Sequencing and Mutation Analysis of *β*-Catenin Gene

All hematoxylin and eosin (H&E) stained slides containing adenocarcinoma components were evaluated. Targeted next-generation sequencing was performed in all 721 patients. The lung cancer-associated targeted genes included *EGFR*, *HER2*, *BRAF*, *KRAS*, *NRAS*, *ALK*, *PIK3CA*, *MET*, *RET*, and *ROS1*. The paraffin blocks with the most abundant morule-like components were sorted out for molecular analysis. The nontumorous components were removed as much as possible based on the morphological examination. The method of DNA extraction, DNA concentration, next-generation sequencing, and data analysis was identical to our previous study [14]. The *β-catenin* gene (*CTNNB1*) exon 3 was amplified by polymerase chain reaction (PCR). The primer pairs were as follows: 5′-GATTTGATGGAGTTGGACATGG-3′(F) and 5′-GCTACTTGTTCTTGAGTGAAGG-3′ (R). PCR amplification conditions were as follows: predenaturation at 98°C for 5 min, 35 cycles of 95°C for 30 s, 55°C for 30 s, and extension at 72°C for 30 s. The PCR data were analyzed using the 3730xl DNA Analyzer (Applied Biosystems, Foster City, USA). Chromas 2.6.6 software were used for analysing the mutations/variations (Technelysium, South Brisbane, Australia).

### 2.4. Statistical Analysis

Statistical procedures were run using the IBM SPSS 20.0 statistical software package for Microsoft Windows. Pearson's *χ*^2^ test was used to assess the association between MLC and EGFR mutation. We used Kaplan-Meier survival analysis to draw the survival curves. The statistical differences between curves were tested by the log-rank test.

## 3. Results

### 3.1. Clinical and Radiological Findings

**Figure 1:**
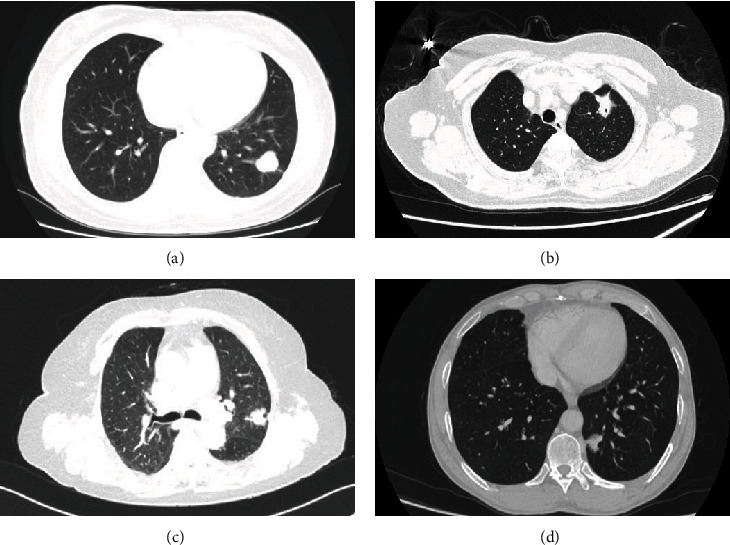
CT imaging of lung adenocarcinomas with MLCs. CT scans showed a solid mass in almost all patients (a–c). A well-defined solid mass in case 1 (a). A spiculated solid mass in case 3 (b). A lobular solid mass in case 16 (c). A mass composed of the central solid component and peripheral ground-glass opacity in case 12 (d).

All clinicopathological records were reviewed, and the relevant results are listed in Table 1. All 721 patients were Chinese people. Of the 20 patients with MLCs, there were 13 women and 7 men. The median age of the 20 patients was 56 years (range from 46 to 70 years). Six patients had a history of smoking. The mean size of the tumor was 2.3 cm (range from 1.0 to 3.4 cm). The patients could manifest chest pain (4 cases) and cough (2 cases). The other 14 patients were asymptomatic. The patients underwent lobectomy (18 cases) or wedge resection (2 cases). The median time of follow-up was 29.3 months (range from 3 to 45 months). CT scans showed a pure solid mass in almost all patients (16/17) (Figures 1(a)–1(c)). In case 12, the mass showed a central solid portion accompanied by peripheral ground-glass opacity and on CT (Figure 1(d)).

### 3.2. Histological Findings

**Figure 2:**
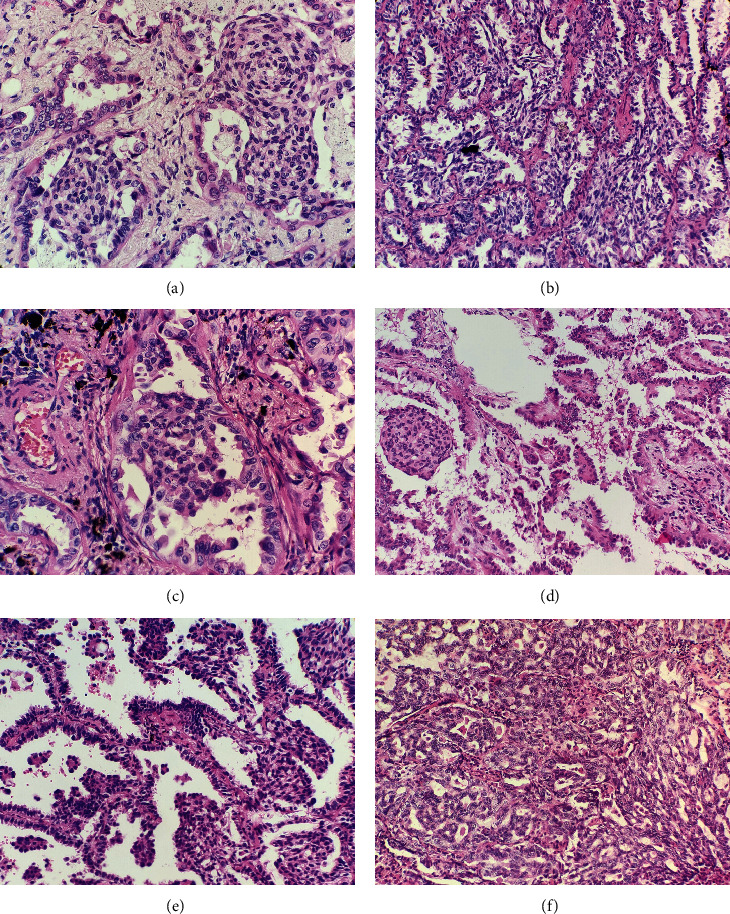
Histological findings of lung adenocarcinomas with MLCs. The MLCs were composed of spindle cells showing a whorled growth pattern, and the spindle cells in the MLCs had a syncytial appearance (a). The MLCs showed a streaming growth pattern with fenestration which was similar to the usual ductal hyperplasia in the breast (b). A few MLCs were epithelioid (c). An MLC in a papillary adenocarcinoma (d). MLCs and micropapillary components in a lepidic adenocarcinoma (e). The transitional region between the cribriform component and the MLC (f).

The morule-like structures were located in the glandular lumens of adenocarcinoma. Most MLCs showed a whorled or streaming growth pattern (Figures 2(a) and 2(b)), and the cells were usually spindle-shaped. Cytoplasmic keratinization and intercellular bridge were not detected in the MLCs. In some cases, the lumens involved by MLC demonstrated a fenestrated growth pattern which was similar to the usual ductal hyperplasia in the breast (Figures 2(a) and 2(b)). A few MLCs demonstrated epithelioid rather than spindle nodules (Figure 2(c)). The cells in MLCs often had a syncytial appearance. The background adenocarcinoma cells frequently demonstrated apical snouting in all cases. Nuclei in most MLCs resembled their adenocarcinoma counterparts. However, in some MLCs, the nuclei appeared slightly smaller and milder than the non-MLC components. The epithelioid cells in MLCs usually showed more atypia than the spindle ones (Figure 2(c)). The mitosis is almost absent in MLCs. Among the 20 cases, 9 were lepidic adenocarcinomas, 8 were acinar adenocarcinomas, 2 were papillary adenocarcinomas (Figure 2(d)), and 1 was a minimally invasive adenocarcinoma. The proportion of MLCs varied from 5% to 50%. A focal micropapillary component (accounting for 5–20%) was observed in 9 cases (45%, Figure 2(e)). A cribriform component (accounting for 10–30%) was observed in 3 cases (15%), and a transitional region between the cribriform component and the MLC could be observed in these cases (Figure 2(f)). Spread through air spaces (STAS) was observed in 5 cases (25%). Six cases (30%) showed a visceral pleural invasion which was demonstrated by elastic staining.

### 3.3. Immunohistochemistry

**Figure 3:**
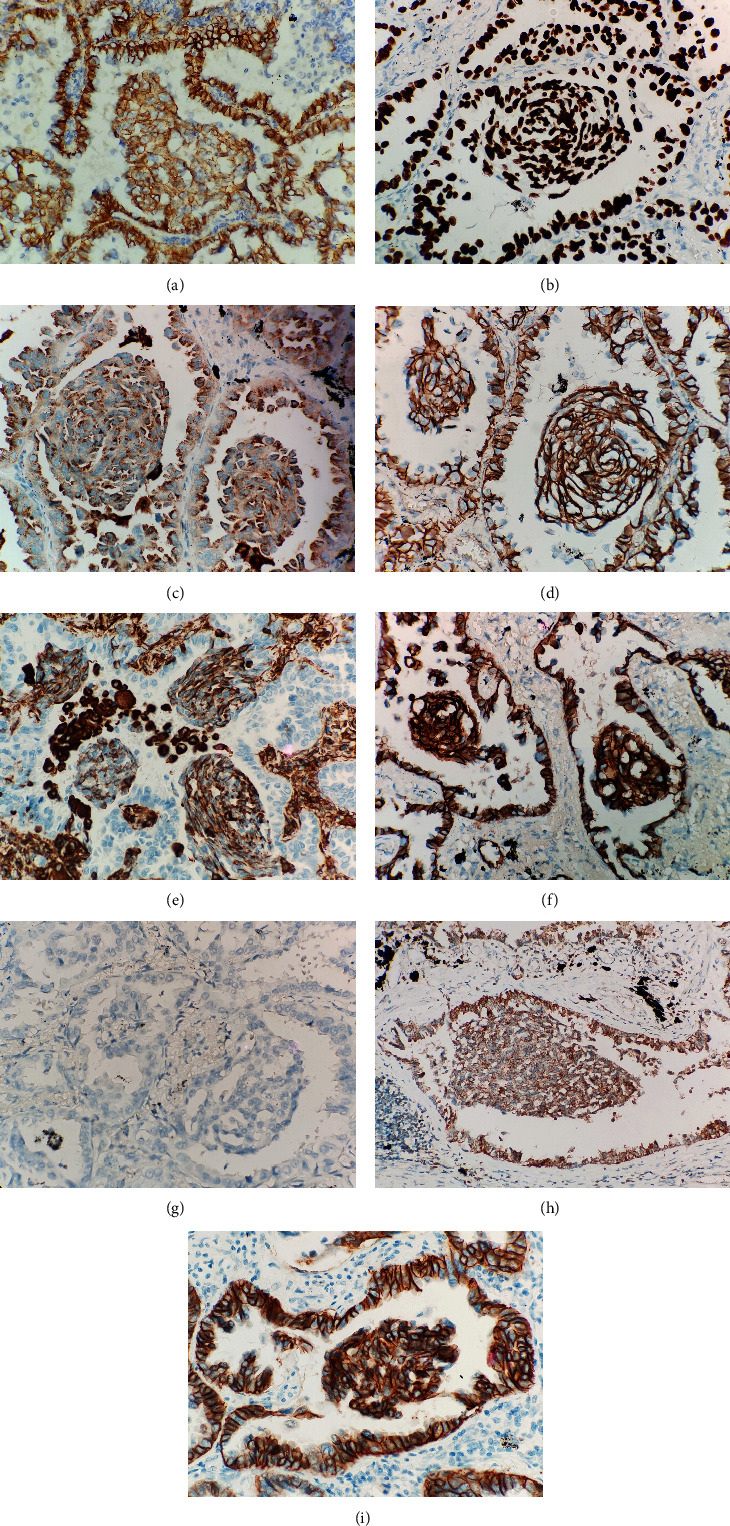
The MLCs were positive for CK7, TTF-1, napsin-A, and E-cadherin (a–d). Vimentin was always positive in the MLCs (e). *β*-Catenin showed membranous staining in all cases (f). The MLCs were negative for p63 (g). ALK immunopositivity showed cytoplasmic granular staining in a case harboring ALK-EML4 fusion (h). Her-2 immunopositivity showed membranous staining in the case harboring HER2 amplification (i).

The immunohistochemical staining result was listed in Table 2. Both MLCs and the adjacent tumor area of the adenocarcinoma were positive for CK7, TTF-1, napsin-A, and E-cadherin (Figures 3(a)–3(d)). Vimentins (V9 and EP21) were positive in the MLCs in all cases and a few non-MLC components (especially in cribriform and micropapillary components) (Figure 3(e)), but abnormal expression (cytoplasmic and nuclear staining) of *β*-catenin was not detected (all showed membranous staining) (Figure 3(f)). The MLCs always showed negative immunoreactivities for CK5/6, p40, p63 (Figure 3(g)), Synaptophysin, chromogranin A, and Cdx-2. The 2 cases with *ALK-EML*4 fusion and the case with *HER*2 amplification were also positive for ALK and Her-2 (Figures 3(h) and 3(i)), respectively. Ki-67 proliferative index in MLCs ranged from 1% to 10%, which was similar to the adjacent tumor areas.

### 3.4. NGS and Sequencing Analysis of *β*-Catenin Gene

**Figure 4:**
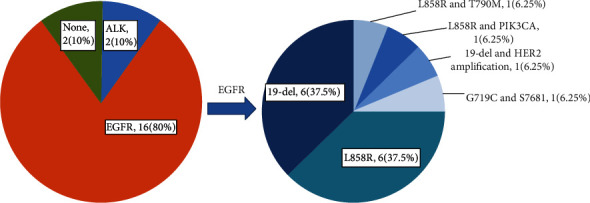
The driver mutation status in lung adenocarcinomas with MLCs.

*EGFR* mutation was detected in 16 patients (80.0%), in which 8 patients showed an exon 21 L858R mutation (1 had a synchronous exon 20 T790M and 1 had a synchronous *PIK3CA* mutation). Exon 19-del was found in 7 patients (1 had a synchronous *HER2* amplification). One patient had synchronous point mutations both in exon 18 and exon 20 (exon18 p.G719C and exon20 p.S768I). *EGFR* mutation was more common in the lung adenocarcinomas accompanied with MLCs than lung adenocarcinomas without MLCs (*χ*^2^ = 4.339, *P* = 0.040). *ALK-EML*4 fusion was detected in two patients (Figure 4). *β-*Catenin gene mutation was not detected in all patients (see Figure S1 in the Supplementary Material).

### 3.5. Impact of MLC on the Prognosis of Lung Adenocarcinoma

**Figure 5:**
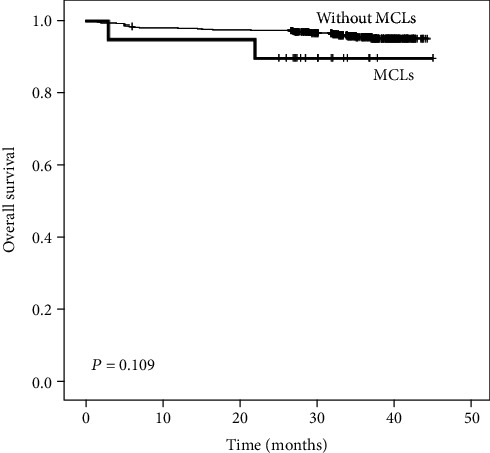
Kaplan-Meier analysis showing the role of MCLs in the prognosis of lung adenocarcinoma. No significant difference in OS was detected between the lung adenocarcinoma with MCLs and without MCL.

Kaplan-Meier survival curves showed that there was no significant difference between lung adenocarcinomas with MLCs and lung adenocarcinomas without MLCs in overall survival (OS) (Figure 5, *P* = 0.109).

## 4. Discussion

MLC, also called nonsarcomatous spindle cell morphology, is a rare architecture in lung adenocarcinoma, accounting for about 0.5%–4.0% of all reported lung adenocarcinomas in the literature [4, 5, 8]. It usually presents in middle-aged to elderly people, and only one case occurred in a patient under 40 [2–8]. MLC accounted for 2.8% of all adenocarcinomas in the present series, with a 1.86 : 1 female/male ratio.

The cells in MLCs usually have a whorled or streaming growth pattern, with a squamoid or sarcomatoid pattern, but no definite squamous differentiation, such as cytoplasmic keratinization or intercellular bridges, has ever been observed. MLCs often arise in well-differentiated adenocarcinomas, such as papillary, lepidic, and acinar patterns [5, 8]. In our study, there were 10 cases of lepidic predominant adenocarcinomas. As we know, lepidic predominant lung adenocarcinoma can appear as a mixed ground-glass opacity on CT [15]. However, only one case in our study appeared as a part-solid mass. This may be related to the MLCs that occupy the alveolar space associated with lepidic adenocarcinoma. A micropapillary component was observed in 50% (10 cases) in our study in contrast to 88% in Tsuta's study [5], although it was usually not a predominant component [5, 8]. As we know, lung adenocarcinomas with micropapillary components tend to have poor prognosis even if the micropapillary component is not predominant [16, 17]. Some lumens involved with MLC showed a fenestrated pattern, which was similar to the usual ductal hyperplasia in the breast. We identified the fenestrated structures from published illustrations, although they were not mentioned in those reports [5, 8]. The fenestrations were peripherally located and tended to be slit-like, in contrast to cribriform adenocarcinoma. A transitional region between the cribriform component and the MLC with a fenestrated pattern was always observed locally in the three cases with cribriform components, and sometimes, it was difficult to distinguish these two components. Two of them showed a worse prognosis (cases 6 and 10). Although lung adenocarcinoma with a cribriform pattern is not a subtype or variant in the WHO classification [1], recent studies have shown that those lung adenocarcinomas involving cribriform components had a worse outcome [18–21]. Chang et al. contended that the MLC was a histological hallmark of aggressive behavior in lung adenocarcinomas. However, many cases (15/17, 88%) showed a micropapillary component in their study [5]. This poor prognosis is probably due to the micropapillary component or cribriform component rather than the MLCs. In our study, we did not find a significant relationship between the MCLs and overall survival of lung adenocarcinoma patients. We do not recommend classifying the MLC as a solid component because of their mild appearance and uncertain indication for aggressive behavior. Pathologists should pay more attention to the micropapillary components and cribriform components in lung adenocarcinoma with MLCs.

Immunohistochemically, MLCs always express alveolar epithelial markers such as TTF-1, CK7, and napsin-A, indicating that the MLCs may originate from alveolar pneumocytes. The neuroendocrine markers and squamous epithelial markers were usually negative, showing that they did not represent a neuroendocrine component, a squamous cell component in an adenosquamous carcinoma or squamous metaplasia. Traditional morules always showed aberrant nuclear/cytoplasmic expression of *β*-catenin due to the activation of the Wnt/beta-catenin signaling pathway [1, 9–13]. In our study, neither *β-catenin* (*CTNNB1*) gene mutation nor aberrant *β*-catenin expression was detected, which indicated that the Wnt/beta-catenin signaling pathway was not involved. Cdx-2, a marker frequently positive in traditional morules in various lesions [22], was always negative in our study. Thus, we believe that MLCs in lung adenocarcinomas and the traditional morules have different etiology. Vimentin was positive in all 20 cases, which contradicted the results reported by Matsukuma et al. [8]. Two different clones of vimentin were made to verify our results, and they were both positive in MLCs. This discrepancy might be ascribed to different experimental conditions. The spindle syncytial appearance and the expression of vimentin make us think of epithelial-mesenchymal transformation. However, epithelial-mesenchymal transformation often arose in poorly differentiated lung cancer, and it usually showed reduced E-cadherin expression [23–25]. These things were not observed in the MLCs. The formation of MLCs may involve an unrevealed molecular process that needs further study.

Lung adenocarcinoma with MLCs has a high rate of *EGFR* mutation, and some researchers believe that the MLCs are a predictor for *EGFR* mutation in lung adenocarcinoma [5]. In our study, 80% (16/20) of lung adenocarcinomas with MLCs harbored an *EGFR* mutation, higher than those adenocarcinomas without MLCs, which was similar to previous research [5]. However, the *EGFR* mutation is not the only genetic alteration in lung adenocarcinomas with MLCs. At present, among all the cases reported including our cases, three cases were associated with *ALK-EML4* fusion [5], and one case was associated with *KRAS* mutation (substitution mutation at codon 12: G12C) [6]. One case harboring coalteration of exon 19 del and *HER2* amplification and one case harboring coalteration exon 21 L858R mutation and *PIK3CA* point mutation were also detected in the current study. Usually, lepidic, acinar, or papillary components are the predominant components in lung adenocarcinomas with MLCs. As we know, *EGFR* mutations were very common in these subtypes of lung adenocarcinomas [26–32]. In our recently published data, among 814 patients with lung adenocarcinoma, *EGFR* gene alteration was observed in 503 (61.8%) patients, and the *EGFR* gene alteration was frequently found in papillary adenocarcinoma (79.8%), acinar adenocarcinoma (72.4%), and lepidic adenocarcinoma (55.3%) [33]. In this way, the frequent *EGFR* mutation in lung adenocarcinomas with MLCs may be due to the lung adenocarcinoma itself rather than the MLCs. Histologically, lung adenocarcinoma with MLCs must be distinguished from low-grade fetal lung adenocarcinoma. The glandular tumor cells in low-grade fetal adenocarcinoma are columnar with clear cytoplasm and lack apical snouting cytoplasm. Due to the activation of the Wnt/beta-catenin signaling pathway by *β-catenin* gene mutation, low-grade fetal adenocarcinoma usually showed nuclear/cytoplasmic *β*-catenin expression.

## 5. Conclusion

The MLCs are usually accompanied by lepidic, acinar, and papillary predominant lung adenocarcinomas. Lung adenocarcinoma with MLCs tend to appear as a solid mass on CT images. The frequent *EGFR* mutation may be due to the lung adenocarcinoma itself rather than the MLCs. In addition to *EGFR*, *ALK-EML4* fusion, *KRAS* mutation, *HER2* amplification, and *PIK3CA* mutation were also found in lung adenocarcinomas with MLCs. The micropapillary components and cribriform components may cause poor prognosis of lung adenocarcinomas with MLCs. Vimentin is always positive in MLCs and is a useful marker to identify the MLCs.

## Figures and Tables

**Table 1:** Clinicopathologic characteristics of lung adenocarcinomas with MLCs.

Case no.	Age (years)/sex/smoking	Location	Size (cm)	Histologic findings					Treatment	TNM stage	Follow-up (months)
				Proportion of MLCs (%)	Predominant component	Other components	STAS	Visceral pleural invasion			
1	50/F/no	LLL	2.2	10	Acinar	Micropapillary	—	—	Lobectomy	T1N1M0	AWT (45)
2	46/F/no	RLL	2.5	15	Acinar	Micropapillary	+	—	Lobectomy	T1N0M0	NET (38)
3	68/F/no	LUL	3.0	20	Acinar	Micropapillary	—	+	Lobectomy	T2N0M0	NET (37)
4	46/F/no	RUL	2.5	5	Acinar	Lepidic/micropapillary	—	—	Lobectomy	T1N0M0	NET (37)
5	48/M/yes	LUL	2.0	5	Lepidic	Acinar	+	—	Lobectomy	T1N1M0	NET (34)
6	66/M/yes	LLL	3.2	15	Lepidic	Papillary/cribriform	—	—	Lobectomy	T2N1M0	DOT(22)
7	55/F/no	RLL	3.1	10	Lepidic	Papillary	—	—	Lobectomy	T2N0M0	NET (33)
8^∗^	51/M/no	RLL	1.2	15	Lepidic	Acinar	—	—	Lobectomy	T1N1M0	NET (32)
9	56/F/no	LUL	1.0	20	Papillary	Lepidic	—	—	Lobectomy	T1N0M0	NET (32)
10	53/M/yes	LLL	2.0	15	Lepidic	Cribriform	—	+	Lobectomy	T2N1M0	DOT (3)
11	68/F/no	LUL	2.5	25	Lepidic	Acinar	—	—	Lobectomy	T1N0M0	NET (30)
12	60/M/no	LLL	2.0	5	Lepidic	Acinar/micropapillary	—	—	Wedge resection	T1N0M0	NET (30)
13	56/M/yes	LLL	3.4	15	Lepidic	Papillary/micropapillary/acinar	+	+	Lobectomy	T2N1M0	NET (29)
14	68/F/yes	RML	1.3	5	Lepidic	Cribriform/micropapillary	+	—	Lobectomy	T1N0M0	NET (28)
15	55/F/no	LUL	2.5	35	Acinar	Micropapillary	+	—	Lobectomy	T1N0M0	Lost to follow-up
16	70/F/no	RUL	2.3	50	Acinar	None	—	+	Wedge resection	T2N0M0	NET (28)
17	61/M/no	RUL	3.0	10	Acinar	Micropapillary	—	—	Lobectomy	T1N1M0	NET (27)
18	48/F/no	LUL	2.8	5	Lepidic	Acinar	—	+	Lobectomy	T2N0M0	NET (27)
19	66/F/yes	LUL	2.5	10	Acinar	Lepidic	—	+	Lobectomy	T2N0M0	NET (27)
20	68/F/no	RLL	1.7	5	Papillary	Lepidic	—	—	Lobectomy	T1N0M0	NET (26)

AWT: alive with tumor (recurrence); DOT: died of tumor; F: female; LLL: left lower lobe; LUL: left upper lobe; M: male; NET: no evidence of tumor; RLL: right lower lobe; RML: right middle lobe; RUL: right upper lobe; STAS: spread through air spaces. ^∗^This case was a minimally invasive adenocarcinoma.

**Table 2:** Immunohistochemical features of adenocarcinoma with MLCs.

Antibody	MLC	Adjacent tumour area
CK7	20 (100%)	20 (100%)
TTF-1	20 (100%)	20 (100%)
Napsin-A	20 (100%)	20 (100%)
E-cadherin	20 (100%)	20 (100%)
Vimentin (V9)	20 (100%)	6 (30%)
Vimentin (EP21)	20 (100%)	6 (30%)
*β*-Catenin		
Membranous	20 (100%)	20 (100%)
Cytoplasmic and nuclear	0	0
CK5/6	0	0
p40	0	0
p63	0	0
Synaptophysin	0	0
Chromogranin A	0	0
Cdx-2	0	0
Ki-67 index	1%-10%	1%-10%

## Data Availability

The data in this paper which were used to support the study analysis are available upon request from the corresponding authors.

## Supplementary Materials

Supplementary Materials Supplementary 1. Figure S1: *β-catenin* gene mutation was not detected.
