# Supplementary material for: Clinicopathological, Radiological, and Molecular Features of Primary Lung Adenocarcinoma with Morule-Like Components
Source: Dis Markers. 2021 Jun 12;2021:9186056. doi: 10.1155/2021/9186056 (PMC8216805; doi:10.1155/2021/9186056)
Supplement: Supplementary Materials — Supplementary 1. Figure S1: β-catenin gene mutation was not detected. [file 9186056.f1.pdf]

Supplementary figure

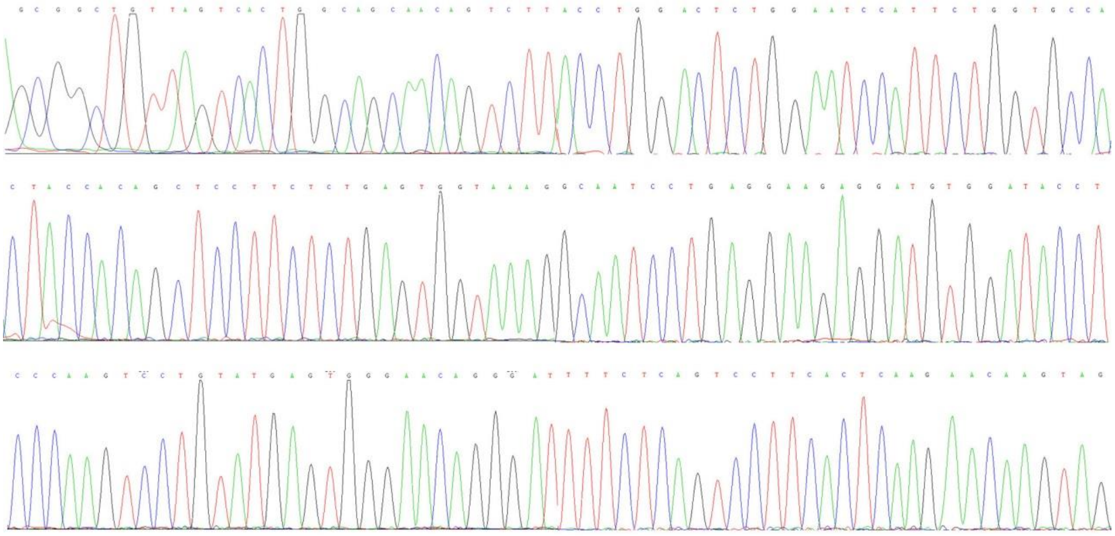

FIGURE S1. The mutations of *β-catenin* gene in Exon 3. No *β-catenin* gene mutation in Exon 3 was detected in all patients.
